# Bifunctional Microcapsules with n-Octadecane/Thyme Oil Core and Polyurea Shell for High-Efficiency Thermal Energy Storage and Antibiosis

**DOI:** 10.3390/polym12102226

**Published:** 2020-09-28

**Authors:** Xianfeng Wang, Chunhong Li, Meihui Wang, Tao Zhao, Wenyao Li

**Affiliations:** 1 College of Chemistry, Chemical Engineering and Biotechnology, Donghua University, Shanghai 201620, China; tim.wang@dhu.edu.cn (X.W.); 2150561@mail.dhu.edu.cn (M.W.); 2 Lutai School of Textile and Apparel, Shandong University of Technology, Zibo 255000, China; 05278@sdut.edu.cn; 3 Key Laboratory of Science and Technology of Eco-Textile, Ministry of Education, Donghua University, Shanghai 201620, China; 4 School of Materials Engineering, Shanghai University of Engineering Science, Shanghai 201620, China

**Keywords:** bifunctional microcapsules, phase change materials, thyme oil, antibiosis, latent heat storage

## Abstract

A new kind of bifunctional microcapsule containing a n-octadecane (OD) and thyme oil (TO) core based on polyurea shell designed for thermal energy storage and antibiosis was prepared successfully through interfacial polymerization. The scanning electron microscopic investigations reveal that the obtained composite microcapsules present the regular spherical morphology and the transmission electron microscopic observations confirm the clear core–shell structure. Morphological and chemical structure analyses prove the successful synthesis of bifunctional microcapsules. Thermogravimetric analysis indicates that the polyurea shell can protect the composite cores effectively. Differential scanning calorimetry examination shows that the bifunctional microcapsules can maintain high thermal storage capacity and the encapsulation efficiency of OD increases with the increase in TO. The supercooling crystallization can be notably suppressed by adding 7 wt.% of n-octadecanol. A study on the release behavior of TO from the bifunctional microcapsules reveals that the Higuchi kinetic model could better fit the TO release profile. The antibacterial results demonstrate that the bifunctional microcapsules can effectively inhibit the growth of *Staphylococcus aureus* and the inhibition rate can reach as high as 99.9% when the mass concentration of microcapsules is over 3 wt.%.

## 1. Introduction

Phase change materials (PCMs) are a class of latent-heat storage materials that possess high thermal storage density in small temperature intervals [1]. However, the leakage problem and obvious volume changes during the phase transition processes limit the use of pristine PCMs. Microencapsulation of PCMs can maintain the macroscopic solid form of PCMs, enlarge the heat exchange area and reduce the interaction between PCMs and the environment [2,3,4]. As a feasible route for resolving the handling difficulty of bulk PCMs, the microencapsulation technique has received increasing attention for over 20 years. Different inorganic and organic polymer film-forming materials have been employed to encapsulate the various types of PCMs. The common inorganic shells cover SiO_2_ [5], TiO_2_ [6] and CaCO_3_ [7] while the usually used organic wall materials include melamine resin [8], acrylic resin [9], polyurea (PU) [10], carbon [11] and Arabic gelatin-gum [12]. The development of microencapsulated PCMs (microPCMs) has promoted the application of PCMs in the area of the textile industry, building, food industry, electronic equipment, utilization of solar energy, and so forth [13,14,15].

In the previous research, many researchers have focused on the principle, materials and process parameters of preparation of microPCMs as well as their properties including heat storage property, heat resistance and heat-conducting property [16,17]. In recent years, the value-added functionalization of microPCMs has aroused the concern of some researchers. The bifunctional microPCMs refer to the microPCMs that possess thermal energy storage ability and other functionalities. Endowing microPCMs with new functions other than thermoregulation provides the potential to expand their application fields and improve the application value [6,18,19,20]. By virtue of the functional diversity of some inorganic materials, several multifunctional microPCMs with new function effectiveness such as magnetic property [20,21], photocatalysis [22], photothermal conversion [23,24,25], photoluminescence [26], and antibacterial property [16,17,18,19] have been developed.

Among these multifunctional microPCMs, antibacterial microPCMs highlight the potential application in thermoregulated textiles including medical textiles, food preservation and packaging, and energy-saving buildings [17]. Take the antibacterial temperature-adaptable fabrics, for example, at least two different functional additives should be applied to the fabrics to achieve both of the features of antibiosis and thermoregulation with traditional materials [27] while the antibacterial microPCMs can provide an easier method. Similar to the other multifunctional microPCMs, the addition of antibacterial performance mainly depends on the selected inorganic materials according to the few open pieces of literature. Li et al. [16] encapsulated n-eicosane using zinc oxide as the wall material and found that the microPCMs obtained good photocatalysis capability and antimicrobial property. Zhang et al. [17] prepared the multifunctional microcapsules based on Ag/SiO_2_ shell and the obtained solution containing 3 wt% of multifunctional microPCMs showed good antimicrobial effectiveness. Wang et al. [18] synthesized a kind of microcapsule with a silver/silica composite shell through the reduction and deposition of silver ions onto the surface of SiO_2_ microPCMs and then they were used to form a PVA hydrogel, which obtained thermoregulation and excellent bactericidal properties. Li et al. [19] obtained a kind of antibacterial microPCMs with lignin as the reducing agent for silver and the resulting microcapsules exhibited high heat enthalpy and good antibacterial activity. However, in this research, the most commonly used antibiotic additive was silver nanoparticles which may have potentially toxic effects on humans and are not environmentally-friendly [28,29].

Natural antimicrobials such as essential oils (EOs) have aroused the increasing concern of researchers and the industrial field because of their safety for mankind and nature in their conventional dosage and the lesser development of resistance under the multiple mechanisms of action [30,31,32]. The microencapsulation technique is an effective alternative route in keeping the stabilization of EOs and provides a controlled release rate of volatile ingredients [33,34]. The published papers have reported evidence of increased antibacterial activity and stability after the EOs are encapsulated when compared with unprotected ones during storage [30,35,36,37]. Nonetheless, the integration of natural antibacterial EOs and microPCMs is insufficient in research. As a type of natural antibacterial agent, thyme oil (TO) possesses the excellent antimicrobial performance, which is closely related with the existence of thymol, carvacrol, eugenol, geraniol and citronellal, especially the former phenolic compounds [33,38,39,40]. Moreover, it can also be used as a co-solvent to dissolve some oil-soluble shell-forming agents and PCMs to improve the compatibility of the oil phase.

Herein, a type of new antibacterial microPCMs containing n-octadecane (OD) and TO composite core materials with PU as the shell was fabricated through interfacial polymerization. n-Octadecane was used as PCM and TO was used as bactericide as well as a co-solvent to dissolve isophorone diisocyanate and OD to improve the compatibility of the oil phase. Meanwhile, the preparation principle was elucidated and the properties of the bifunctional microcapsules including morphology, chemical structure, thermal performance, release behavior and antibacterial effectiveness were also studied. The resultant antibacterial microPCMs possess applicational prospects in thermoregulated textiles and food preservation and packaging.

## 2. Materials and Methods

### 2.1. Materials

Isophorone diisocyanate (IPDI, analytical pure) was purchased from TCI (Shanghai) Chemical Industry Co., Ltd., China. n-Octadecane (OD, 90wt.%) was obtained from Tianjin Alfa Aesar Company, China. Hexamethylene diamine (HMDA, analytical pure), Arabic gum (GA, biochemical reagent) and agar powder (biochemical reagent) were supplied by Sinopharm Chemical Reagent Co., Ltd., China. Thyme oil (TO, industrial purity) was provided by Jiangxi Cedar Natural Medicinal Oil Co., Ltd., China. Tryptone (biochemical reagent) and yeast extract (biochemical reagent) were supplied by Thermo Fisher Scientific (China) Co., Ltd. *Staphylococcus aureus* (ATCC6538) was purchased from Nanjing Bianzhen Biological Technology Co., Ltd., China. All the chemicals were used as received.

### 2.2. Preparation of Antibacterial MicroPCMs

The antibacterial microPCMs were synthesized through the interfacial polymerization method, and the detailed process is as follows: First, the homogeneous water phase was obtained by dissolving gum arabic (GA) into deionized water. OD, TO, and IPDI were mixed uniformly to prepare the oil phase. Then, the oily mixtures were slowly poured into the water phase and emulsified for 5 min at a shearing rate of 7200 rpm using an emulsification machine (T25, IKA Group, Staufen, Germany). Afterward, the O/W emulsion was transferred into a three-necked flask and heated to 35 °C while stirring. Then the temperature was elevated to 60 °C after HMDA solution (15 wt.%) was slowly added into the reaction system and the mixed suspension was continuously stirred for 2 h. After the microcapsule suspension was washed and filtered, the resultant cake was dried for 24 h with a lyophilizer (FD-1A-50, Shanghai Titan Scientific Co., Ltd., China) to obtain the final product. Different antibacterial microPCMs were prepared by changing the ratio of OD/TO and the detailed recipes of the synthesis process are shown in Table 1.

### 2.3. Characterization

#### 2.3.1. Morphology Analysis

The morphology of the microcapsules was observed with a scanning electron microscope (SEM, TM-1000, Hitachi Inc., Tokyo, Japan). The transmission electron microscope (TEM, JEM-2100, JEOL Ltd., Tokyo, Japan) was employed to confirm the core–shell structure of microcapsules.

#### 2.3.2. Chemical Structure Analysis

Fourier transform infrared spectrometer (FTIR, Spectrum-Two, PerkinElmer Ltd., Waltham, MA, USA) was employed to test the infrared spectrum of the products to identify their chemical structures. 

#### 2.3.3. Thermal Stability Test

The thermal gravimetric analyzer (TGA, TG 209 F1, Netzsch GmbH, Selb, Germany) was used to test the heat resistance performance of the samples with the protection of nitrogen. The weight of each sample was approximately 5 mg and the heating rate was set at 10 °C/min from 50 °C to 600 °C. 

#### 2.3.4. Thermal Storage Property

The heat storage properties of different microcapsules were investigated by employing a differential scanning calorimeter (DSC, DSC 204 F1, Netzsch GmbH, Selb, Germany) between −5 °C and 50 °C under the protection of nitrogen. The sample mass was about 3 mg and the temperature rising and cooling rates were set at 5 °C/min.

#### 2.3.5. Encapsulation Efficiency and Release Behavior of TO from Microcapsules

UV-visible spectrophotometry (UV3600PLUS, Shimadzu Ltd., Kyoto, Japan)was used to test the encapsulation efficiency of TO by following the reported methods [41,42]. The detailed process was as follows: firstly, a certain amount of TO dissolved in n-hexane to form standard samples with different concentrations. Then, the absorbancy of standard solutions was tested after the maximum absorption wavelength was determined. Then, the linear relationship of the concentration of TO and the absorbancy was built up. Finally, a certain amount of microcapsule slurries was added into n-hexane with magnetic stirring for 60 seconds and the unencapsulated TO was extracted into the n-hexane. The supernatant solution was separated and its absorbancy was determined by spectrophotometry. Then the mass of free TO (*m_f_*) was calculated and the encapsulation efficiency (*E_TO_*) was determined according to Equation (1), where *m_i_* represents the initial additional amount of TO.

(1) $$E_{TO}=\frac{m_{i}-m_{f}}{m_{i}}\times 100\%$$

The volatile TO can diffuse out of the composite microcapsules through the walls slowly while the nonvolatile OD cannot. According to the reported methods [42,43], the release behavior of TO was tested by the weighing method. A certain mass of dry microcapsules and TO, whose mass should be equal to the mass of TO contained in the control microcapsules, were placed in an oven at 50 °C. Then the samples were weighed at a fixed time and the mass changes of the microcapsules represented the cumulative released weight of TO.

#### 2.3.6. Antibacterial Test

The standard plate count method was adopted to evaluate the antibacterial effectiveness of the microcapsules [17,19,36]. According to the related literature [27,39,44], TO has shown an excellent antibacterial effect to both *Escherichia coli* and *Staphylococcus aureus*, and then, *Staphylococcus aureus* was used here to evaluated the antibacterial activity of microcapsules of different concentrations. Briefly, the inoculated bacterial suspensions were obtained by cultivating the bacteria in the Luria–Bertani medium for 24 h at 37 °C. Afterward, different amounts of TO-loaded microcapsules were mixed with 50 mL of sterilized phosphate-buffered saline (PBS) solution and diluted bacterial suspension to achieve a series of suspensions containing different concentration of the microcapsules. The suspension without microcapsules was used as blank control. Then, the suspensions were incubated for 18 h at 37 °C. The cultivated mixtures were diluted using a 10-fold dilution method and incubated at 37 °C for another 24 h after being placed on the Luria–Bertani agar plate. Then, the number of bacterial colonies in different zones was counted. The antibacterial performance was represented with the inhibition rate (*Y*) calculated according to Equation (2), where *A* is the number of bacterial colonies of the control group and *B* is that of the sample group.

(2) $$Y=\frac{A-B}{A}\times 100\%$$

## 3. Results and Discussion

### 3.1. Preparation and Formation Mechanism of Antibacterial microPCMs

**Figure 1:**
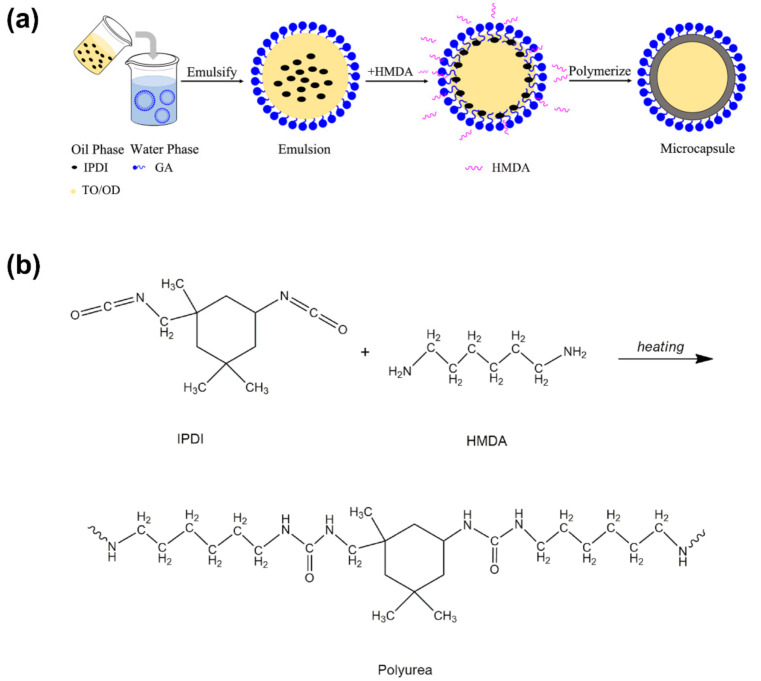
Schematic illustration of the preparation of microcapsules with antibacterial and thermoregulated function (**a**) and schematic diagram of the formation of polyurea (PU) polymer (**b**).

The preparation of antibacterial microPCMs can be elucidated with the interfacial polymerization process. Figure 1a shows the illustration of the forming process of antibacterial microPCMs. TO and OD were used as core materials and the oil phase in which the reaction monomer IPDI was dissolved as an oil-soluble monomer. After the oil phase was poured into the GA aqueous solution, the oil phase was dispersed into small oil droplets under shearing action. Then, the emulsifier GA quickly absorbed onto the oil–water interfaces with its hydrophobic segments oriented into the oil phase and hydrophilic segments oriented into the water phase, resulting in a decrease in interfacial tension. In addition, the steric hindrance created by GA molecules can also prevent the aggregation of oil droplets and ensure the stability of O/W emulsion. The other monomer, HMDA, was dissolved in water as a water-soluble monomer and it moved to the surface of oil droplets once it was added into the reaction system. When HMDA encountered IPDI on the emulsion interface, the highly reactive isocyanate groups and amino groups reacted rapidly to form carbamido groups. With the decrease in reactive monomers at the interfaces, HMDA and IPDI migrated from different phases to the interfaces under the driving force of the concentration gradient to participate in the polymerization, forming the PU polymer as the wall materials (see Figure 1b). Finally, both of the core materials were coated and protected by the shell.

### 3.2. Morphology of the Microcapsules

**Figure 2:**
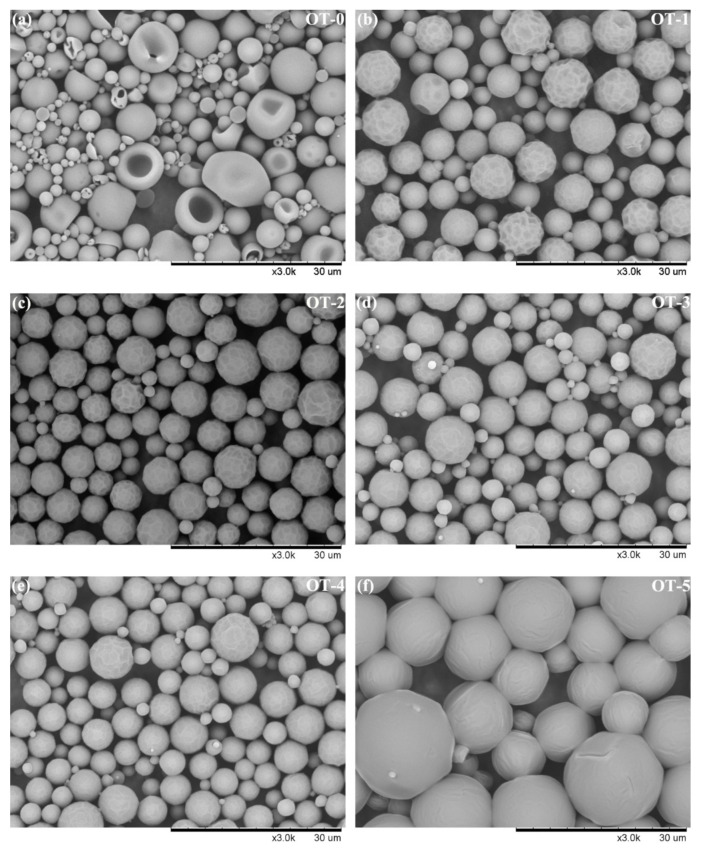
SEM images of composite microcapsules with different n-Octadecane (OD) to thyme oil (TO) ratio: (**a**) 0:12; (**b**) 3:1; (**c**) 4:1; (**d**) 5:1; (**e**) 6:1; (**f**) 12:0.

Figure 2 presents the SEM images of composite microcapsules prepared with various OD to TO ratio. It is visible that sample OT-0 and OT-5 (Figure 2a,f) have notably different morphologies with the other samples. Obvious collapse and potholes on the surface of sample OT-0 without OD can be observed, which is possibly due to the fast diffusion of TO to the outside under the drying process. After the addition of OD, the distinct potholes of as-prepared samples transformed into slight dimple-like potholes as shown in Figure 2b–e (sample OT-1 to OT-4). All these four samples exhibit a more regular spherical shape. The superficial potholes disappear when the nonvolatile and nonpolar OD becomes the sole core material. However, as for the particle size, the diameter of sample OT-5 is larger than that of the other samples markedly while many tiny capsules are existing in sample OT-0. The possible reason is that some amphipathic chemicals such as geraniol in TO can act as co-emulsifier and help to stabilize the emulsion and promote the decrease in particle size. Overview of the morphology and particle size results indicates the composite microcapsules containing dual-core materials have a more regular appearance with proper particle diameter than the microcapsules containing the sole OD or TO core.

**Figure 3:**
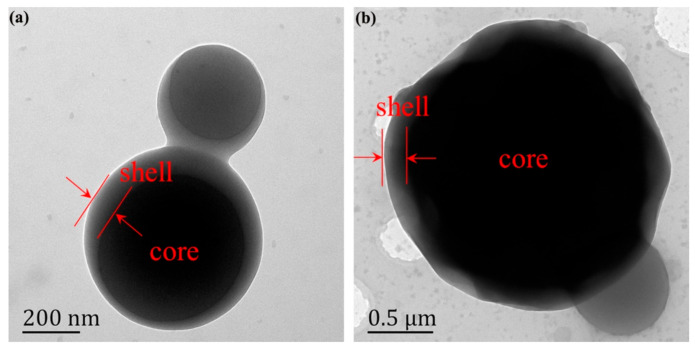
(**a**,**b**) Transition electron microscope (TEM) images of the composite microcapsules (sample OT-2).

The core–shell structural characteristic of the composite microcapsules (sample OT-2) was observed by TEM. It is evident in Figure 3 that the antibacterial microPCMs have a clear core–shell structure. The grain diameter analysis software Nano Measurer was employed to measure the particle diameter and average wall thickness. As shown in Figure 3a, the diameter of the bigger capsule without any wrinkles on the surface is approximately 631 nm and its average shell thickness is approximately 63 nm. As for the image in Figure 3b, the particle size of the microcapsule is approximately 2.2 μm and its average wall thickness is about 149 nm. It is interestingly noted that there exist obvious wrinkles on its surface, which is in agreement with the SEM results.

### 3.3. Chemical Structure of the Microcapsules

**Figure 4:**
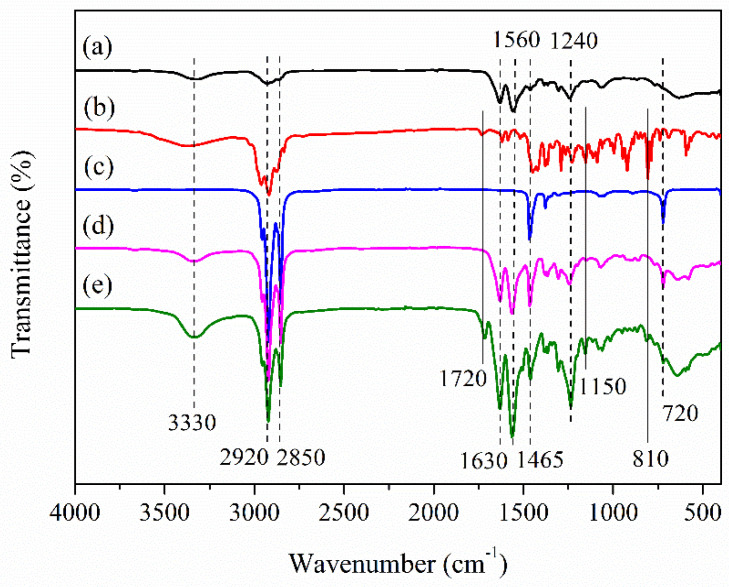
Fourier transform infrared spectrometer (FTIR) spectra of (**a**) PU polymer, (**b**) TO, (**c**) OD, (**d**) microPCMs and (**e**) antibacterial microPCMs.

The chemical structures of PU polymer, TO, OD, microPCMs and antibacterial microPCMs are analyzed by FTIR (Figure 4). As for the spectrogram of PU resin, the absorption peak located near 3330 cm^−1^ belongs to the stretching vibration of N-H bond, and the peak near 1630 cm^−1^ is the stretching vibration absorption peak of the C=O bond in carbamido. The absorption near 1560 cm^−1^ is caused by the bending vibration of N–H bond and the absorption peak near 1240 cm^−1^ is ascribed to the stretching vibration of C–N bond. As for OD, the absorption peaks located near 2920 cm^−1^ and 2850 cm^−1^ correspond to the asymmetric and symmetrical stretching vibration of C–H bond. The peak near 1465 cm^−1^ belongs to the shear bending vibration absorption peak of C–H bond in –CH_2_, and the absorption peak near 720 cm^−1^ is assigned to the in-plane swing vibration absorption peak of –CH_2_. It can be found from Figure 4d that the spectrum of microPCMs includes both of the characteristic absorption peaks of OD and PU resin. Compared with microPCMs, three new characteristic peaks (indicated by solid lines in Figure 4) appear in the spectrum of antibacterial microPCMs and they are also observed in the spectrogram of TO. The peak near 1720 cm^−1^ results from the stretching vibration of the C=O bond of ketones contained in TO. The absorption peak near 1150 cm^−1^ is ascribed to the stretching vibration of C–O bond of alcohol in TO. The absorption peak around 810 cm^−1^ is attributed to the out-of-plane bending vibration absorption peak of the C–H bond on the benzene ring of the phenolic substances in TO. Based on the analysis of morphology and chemical structure, it can be concluded that bifunctional microcapsules were synthesized successfully.

### 3.4. Thermal Stability of the Microcapsules

**Figure 5:**
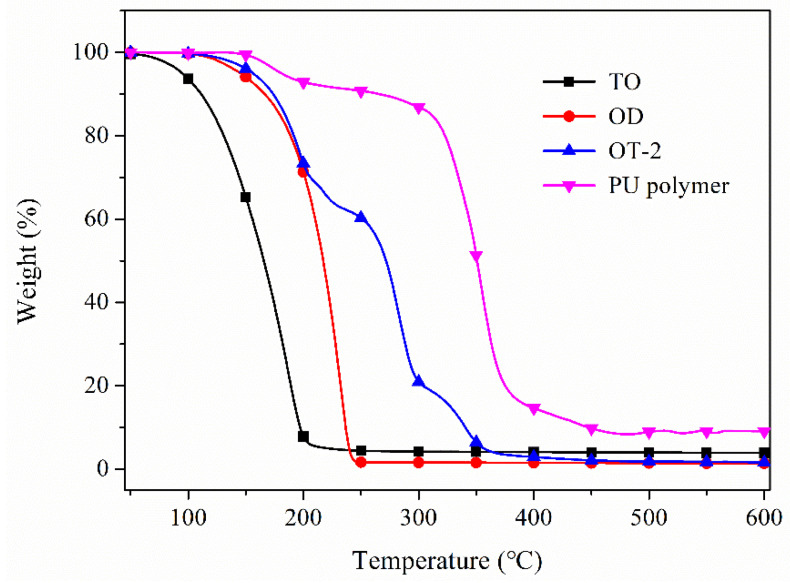
Thermal gravimetric analyzer (TGA) curves of TO, OD, composite microcapsules (sample OT-2) and PU polymer.

The TGA thermograms of TO, OD, sample OT-2 and PU polymer are shown in Figure 5. It can be observed from the TGA curve of TO that it begins to experience weight loss at 55 °C and evaporates completely when the temperature rises to 200 °C, indicating that TO has strong volatility. The pure OD also experiences a one-stage weight loss process on account of the evaporation of OD and shows better thermal stability than TO. With regard to the composite microcapsule (sample OT-2), it exhibits a three-step degradation pattern. The first-stage weight loss process at 50–203 °C is ascribed to the partial volatilization of TO and OD. The second main weight decrease from 203 to 294 °C was caused by the diffusion and release of the residual core materials through the ruptured shells. The weight loss during the third stage in the range of 294–355 °C resulted from the thermal degradation of the wall materials. *T_d5_* and *T_d10_* refer to the temperature when the weight decrease rate reaches 5% and 10%, respectively. The two indices are usually adopted to analyze the heat resistance and the corresponding data are collected in Table 2. *T_d5_* and *T_d10_* of all the composite microcapsules are higher than those of core materials, suggesting that PU polymer can protect the composite cores effectively.

**Figure 6:**
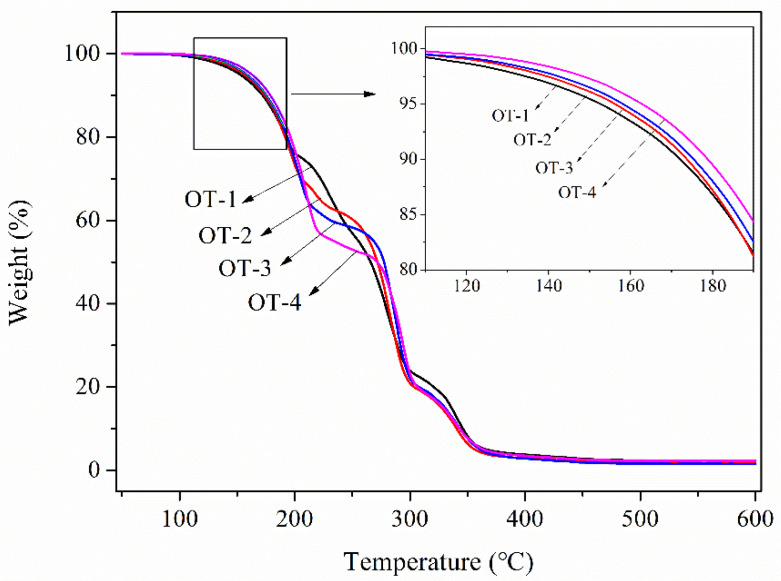
TGA curves of composite microcapsules with different OD to TO ratio.

The TGA curves of the composite microcapsules with different OD to TO ratios are also plotted in Figure 6. As seen from Figure 6, four samples obey almost the same weight loss pattern but own varied weight-loss temperature and weight-loss rate. The thermal parameters of each sample are recorded in Table 2. From sample OT-4 to OT-1, *T_d5_* and *T_d10_* decrease gradually with the increase in TO in core materials. The main reason for this phenomenon is that TO is extremely volatile compared to OD and the initial weight loss of the first-step degradation is primarily ascribed to the evaporation of TO. The higher the TO concentration in the cores, the easier for the microcapsules to experience weight decrease. The first-step weight loss rate of samples also decreases with the increase in TO, indicating the second-step weight decrease starts earlier and earlier. This is because the volatilization of TO will increase the pressure in the microcapsules. The high content of TO in cores results in the rupture of the capsules more easily.

### 3.5. Heat Storage Performance of the Microcapsules

**Figure 7:**
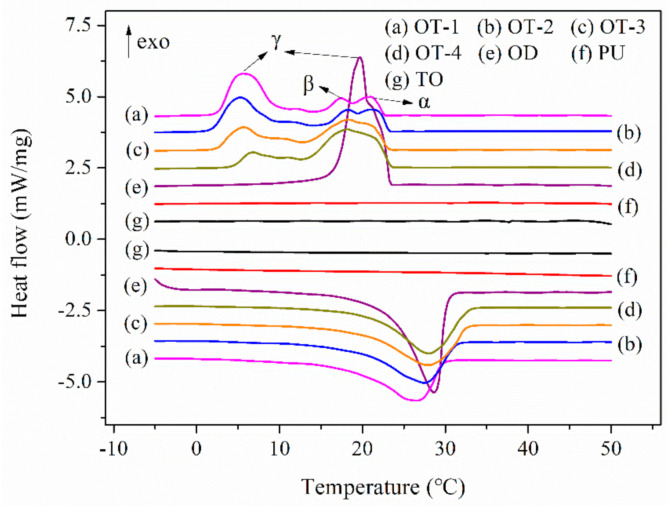
Differential scanning calorimeter (DSC) curves of composite microcapsules, PU polymer, OD and TO.

Thermal storage properties of the resultant microcapsules, PU polymer, OD and TO were studied by the DSC. The obtained DSC curves are displayed in Figure 7, and the corresponding thermal parameters are recorded in Table 3. It can be observable from Figure 7f,g that both PU polymer and TO experience no phase transition in the range of −5 °C and 50 °C. As for pure OD, it shows a single melting peak at 28.6 °C in the temperature-rise period but demonstrates two exothermic peaks (peak α and γ) at 20.9 °C and 19.7 °C during the temperature-fall period. According to the reports in the literature, the formation of peak α is caused by the heterogeneous nucleation of liquid to the rotator phase change and the peak γ is ascribed to the homogeneous nucleation of liquid to the crystal transition [45,46]. With respect to the bifunctional microcapsules, all these four composite microcapsules display nearly the same phase change behaviors with OD while their fusion peak temperatures decrease from 28 °C to 26.5 °C with the increase in TO. This could be explained by the fact that TO can act as a co-solvent to dissolve PCMs, thus accelerating the melt of OD. As far as the crystallization property is concerned, there exist three exothermic peaks labeled α, β, and γ during the crystallization of the microcapsules. The new peak results from the rotator-triclinic phase change [46]. It is notable that the peak γ of OD moves to the low-temperature region after being coated. All the microcapsules exhibit supercooling phenomena just as reported in many works of literature [46,47], resulting from the decrease in the nucleation site in each microcapsule [46].

Thermal storage capacity is an important parameter for heat energy storage materials. The bulk OD exhibits good heat storage capability with melting and crystallizing enthalpies of 214.2 and 215 J/g, respectively (Table 3). However, both the melting and crystallizing enthalpies of the composite microcapsules show a significant decrease due to the fact that PU polymer shell undergoes no phase changes. Furthermore, as presented in Table 3, the heat enthalpies of microcapsules demonstrate a decrease with the increase in the dosage of TO, which is attributed to the decrease in the ratio of OD in composite cores. Nevertheless, all the melting enthalpies of four microcapsules are above 130 J/g, which is acceptable in practical use [18]. The encapsulation efficiency (*E_OD_*) of the composite microcapsules was calculated based on Equation (3), where *m* refers to the final mass of products, *m_0_* represents the initial weight of OD, and *ΔH_m_* and *ΔH_0_* refer to the melting enthalpies of the products and OD, respectively. It is noted that increasing the content of TO in cores improves the encapsulation efficiency of OD to some extent, indicating the addition of TO benefits the encapsulation process.

(3) $$E_{OD}=\frac{m\times \Delta H_{m}}{m_{o}\times \Delta H_{o}}\times 100\%$$

**Figure 8:**
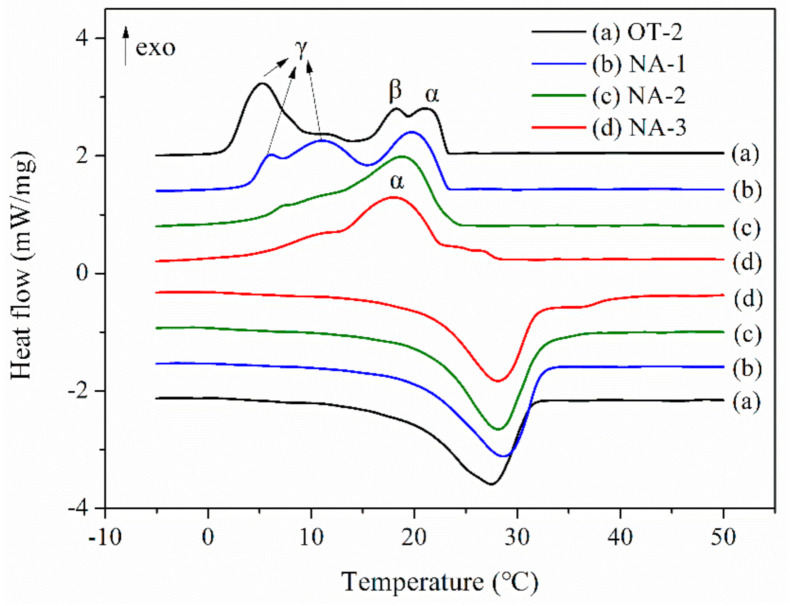
DSC curves of composite microcapsules with n-octadecanol.

As a type of nucleating agent, n-octadecanol was used to suppress the supercooling of the microcapsule (sample OT-2). DSC curves of composite microcapsules with octadecanol are depicted in Figure 8 in which NA-1, NA-2 and NA-3 refer to the microcapsules containing 4 wt.%, 7 wt.% and 10 wt.% of n-octadecanol, respectively. As shown in Figure 8, the addition of the nucleating agent has little effect on the melting behaviors of microcapsules. The melting enthalpies of three microcapsules are 141 J/g, 139.8 J/g and 137 J/g, which is similar to that of sample OT-2 (138.7 J/g), suggesting that the addition of n-octadecanol does not lead to the compromise of thermal capacity [47]. Concerning the crystallizing behavior, there are three exothermic peaks on the DSC crystallization curve of OT-2. Peak γ of NA-1 shifts to higher temperature after the addition of 4 wt.% n-octadecanol while its peak area of low-temperature crystallization is still comparatively large. When the content of n-octadecanol increases to 7 wt.%, it is worth noting that the ratio of the peak area of peak α shows a significant increase from 42.1% to 77.9% in comparison to NA-1, implying the significant suppression of supercooling. This is because the solidification temperature of n-octadecanol is higher than OD and it will solidify firstly during the cooling stage and become the crystal nucleus for the crystallization of OD in composite cores [47], accelerating the nucleation of OD. Therefore, the peak α in NA-2 is ascribed to the heterogeneous nucleation process of liquid to the triclinic phase change. Additionally, the peak area of peak α shows a merely slight enhancement when the dosage of n-octadecanol further increases from 7 wt.% to 10 wt.%. Hence 7 wt.% of n-octadecanol can effectively improve the supercooling crystallization.

### 3.6. Encapsulation Efficiency and Release Behavior of TO from Microcapsules

**Figure 9:**
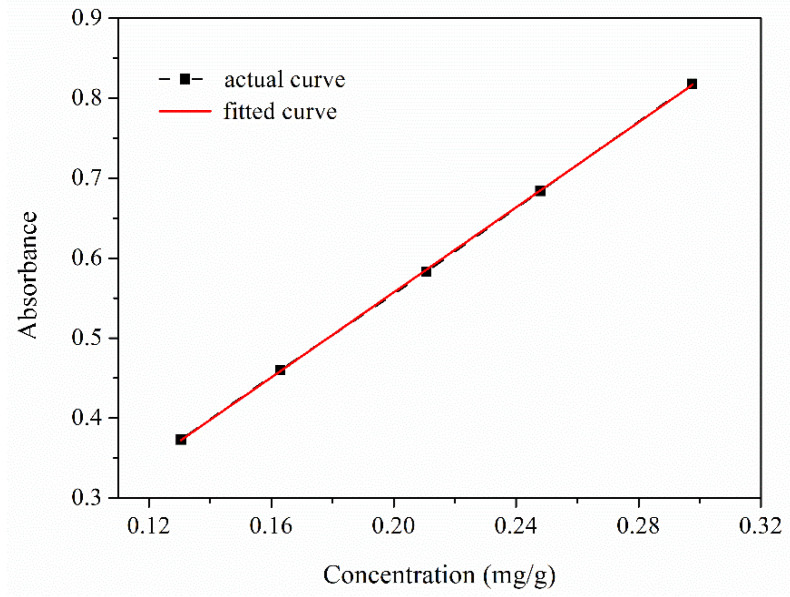
Variation curve of absorbance with the concentration of TO in n-hexane.

Sample OT-2 was selected to calculate the encapsulation efficiency of TO and analyze the release behavior of TO from composite microcapsules. The variation curve of absorbance with the concentration of TO in n-hexane has been plotted in Figure 9 (actual curve), and linear fitting was adopted to analyze the variation and the corresponding fitted curve was also presented in Figure 9. The linear relationship between the concentration of TO (*x*) and absorbance (*y*) can be expressed as *y* = 2.6582 *x* + 0.0255 (*R*^2^ = 0.9999). Then, the concentration of free TO in sample OT-2 was obtained by the fitted equation, thus determining the encapsulation efficiency of TO (*E_TO_*). The *E_TO_* can reach 96.8%, indicating the good encapsulation of TO by PU resin.

**Figure 10:**
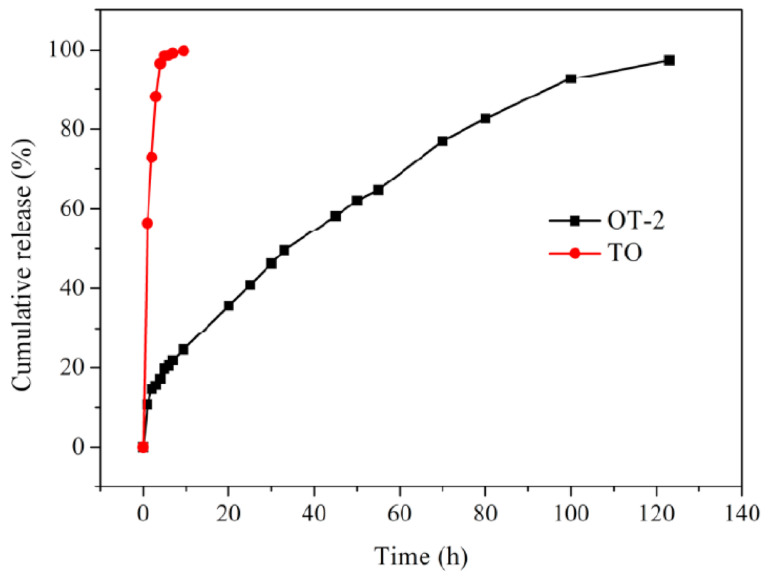
Release curves of TO and composite microcapsules (sample OT-2) at 50 °C.

The release behavior of TO through the shell of composite microcapsules was studied by using the analytical balance, and the release curves of TO and sample OT-2 are depicted in Figure 10. As shown in the release profile of TO, TO evaporates very quickly at 50 °C and the cumulative release rate of TO reaches as high as 98.6% after 5 h while that of composite microcapsules is merely 19.8%. TO encapsulated in sample OT-2 can release slowly and continuously over 120 h at 50 °C, suggesting the composite microcapsules possess the durable sustained-release ability. It can be observed from the release curve of microcapsules that TO releases out of the capsules relatively fast within the first 5 h. This should be ascribed to the initial quick volatilization of TO located near the internal surface of the polymer shell [36]. Afterward, the volatilization of TO from the microcapsules becomes a little slow and steady from 5 h to 70 h. The release rate of TO further decreases after 70 h because of the reduction in concentration difference of TO across the microcapsule shells.

Four dynamical models (as shown in Table 4) were selected to analyze the TO release profile and disclose the release mechanism. The equations of these four models and the matching fitting results are summarized in Table 4. In the above four equations, *Q* refers to the cumulative release rate and *t* represents releasing time. The correlation coefficient (*R*^2^) derived from Higuchi kinetic model is higher than that of the others. Therefore, the equation of the Higuchi kinetic model meet the experiment results very well, suggesting the release of TO from composite microcapsules follows the diffusion mechanism.

### 3.7. Antibacterial Activity of the Microcapsules

**Figure 11:**
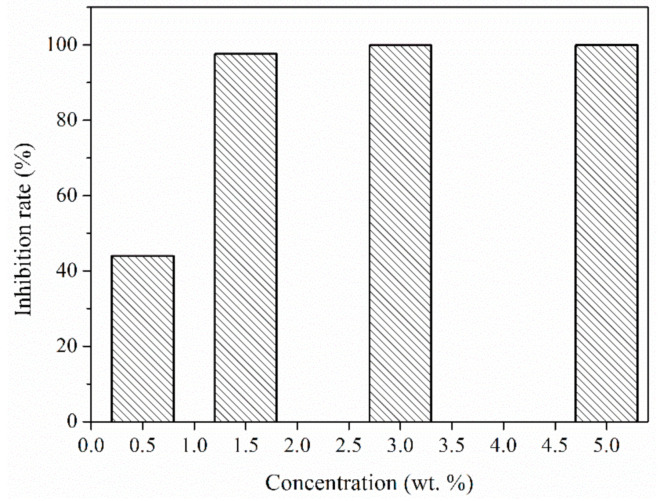
The antimicrobial activities of composite microcapsules (sample OT-2) with different concentrations.

A series of the bacteria solution containing sample OT-2 with different concentrations was prepared and a standard plate count method was adopted to evaluate the antibacterial performance. Figure 11 demonstrates the antibacterial activities of the composite microcapsules with different concentrations to *Staphylococcus aureus*. The antibacterial microPCMs show a weak antibiosis effect with the inhibition rate of 43.95% when the mass concentration is 0.5 wt.%, while it exhibits strong antibacterial performance with the inhibition rate of 97.60% after the concentration rises to 1.5 wt.%. The antibacterial rate can reach as high as 99.9% when the mass concentration of microcapsules is over 3 wt.%. This can be explained by the increase in TO released from the microcapsules within the same time after the increase in mass concentration. The antibacterial activity of composite microcapsules is highly associated with the content of TO in the system. TO enters into the bacteria solutions containing antibacterial microPCMs through diffusion and contacts with *Staphylococcus aureus* and kills the bacteria accordingly during the process of shaking and incubation. The antibacterial mechanism of TO has been investigated by some researchers [39,40,44,48]. The antibiosis performance is attributed to the combined action of various constituents such as thymol, carvacrol, geraniol and eugenol, among which thymol and carvacrol possess more excellent antimicrobial activity [40,44]. It has been reported that thymol and carvacrol can combine with protein in the cytomembrane of bacterial cells with the aid of hydrophobic force and hydrogen-bond interaction and thus change the membrane permeability [48]. In addition, they can also dissolve in the phospholipid bilayer and increase the penetrative performance of the membrane and lead to the leakage of ions and nucleoid in the cytoplasm, thus causing the death of bacteria [49].

## 4. Conclusions

A series of antibacterial microPCMs containing OD and TO dual-core materials based on PU shell was synthesized by interfacial polymerization in this paper. The obtained composite microcapsules present a very regular appearance compared with microcapsules containing the sole TO core and possess smaller particle diameter in comparison to microPCMs based on SEM images. The clear core–shell structure is revealed by TEM observation. Morphological and chemical structure analyses confirm the successful preparation of antibacterial microPCMs. The composite cores can be effectively protected by the PU polymer. DSC analyses demonstrate that the composite microcapsules possess the high thermal capacity and the increasing encapsulation efficiency of OD with the increase in the content of TO. An amount of 7 wt.% of n-octadecanol can effectively suppress the supercooling crystallization. The composite microcapsules exhibit high encapsulation efficiency for TO and possess the durable control–release ability. The release of TO from microcapsules obeys the Higuchi kinetic model. The antibacterial activity of composite microcapsules to *Staphylococcus aureus* increases with the increase in the dosage of microcapsules. The inhibition rate can reach as high as 99.9% when the mass concentration is over 3 wt.%. The bifunctional microcapsules with antibacterial activity and thermoregulated ability are shown to have potential applications in smart textiles and food preservation and packaging.

## Figures and Tables

**Table 1:** The detailed recipes of the synthesis process of different antibacterial microencapsulated phase change materials (microPCMs).

Sample	GA (g)	OD (g)	TO (g)	IPDI (g)	HMDA (g)
OT-0	1.00	0.00	12.00	1.89	1.11
OT-1	1.00	9.00	3.00	1.89	1.11
OT-2	1.00	9.60	2.40	1.89	1.11
OT-3	1.00	10.00	2.00	1.89	1.11
OT-4	1.00	10.29	1.71	1.89	1.11
OT-5	1.00	12.00	0.00	1.89	1.11

**Table 2:** The TGA results of pure cores and various composite microcapsules.

Sample Code	*T_d5_* (°C)	*T_d10_* (°C)	First-Stage Weight Loss Rate (%)
TO	94.8	111.2	96.12
OD	145.4	166.4	96.75
OT-1	152.7	172.2	22.90
OT-2	156.3	173.8	28.74
OT-3	158.2	175.5	35.32
OT-4	162.9	178.9	41.83

**Table 3:** The thermal parameters of pure OD and various composite microcapsules.

Sample Code	Melting Process		Crystallization Process				*E_OD_* (%)
	*T_m_* (°C)	*ΔH_m_* (J/g)	*T_α_* (°C)	*T_β_* (°C)	*T_γ_* (°C)	*ΔH_c_* (J/g)	
OD	28.6	214.2	20.9	-	19.7	215.0	-
OT-1	26.5	133.1	21.0	17.4	5.7	138.0	85.3
OT-2	27.5	138.7	21.0	18.3	5.3	145.1	83.7
OT-3	28.0	146.1	21.2	18.4	5.6	149.0	82.6
OT-4	28.0	154.2	-	18.1	6.8	156.7	82.0

*Note: T_m_*: The peak temperature of melting peak; *ΔH_m_*: Melting enthalpy; *T_α_*, *T_β_* and *T_γ_*: The peak temperature of crystallization peak α, β and γ; *ΔH_c_*: Crystallizing enthalpy.

**Table 4:** The fitting results of the release profile of composite microcapsules.

Model Type	Equation of Model	*R* ^2^
Zero-order dynamical model	*Q* = 0.7872 *t* + 16.2661	0.9438
First-order dynamical model	ln(100-*Q*) = −0.0252 *t* + 4.6355	0.9470
Higuchi dynamical model	*Q* = 9.0098 *t*^1/2^ − 1.0517	0.9944
Korsmeyer–Peppas dynamical model	*Q* = 9.7521 *t*^0.4476^	0.9853
